# Antitumor Effects of Evodiamine in Mice Model Experiments: A Systematic Review and Meta-Analysis

**DOI:** 10.3389/fonc.2021.774201

**Published:** 2021-11-09

**Authors:** Cong Yin, Jing Cheng, Hongbing Peng, Shijun Yuan, Keli Chen, Juan Li

**Affiliations:** Hubei Province Key Laboratory of Traditional Chinese Medicine Resource and Chemistry, Hubei University of Chinese Medicine, Wuhan, China

**Keywords:** evodiamine, tumor, systematic review, meta-analysis, mice model experiments

## Abstract

**Background:**

Evodiamine (EVO), an alkaloid extracted from the traditional Chinese medicine *Euodia rutaecarpa*, plays an important role in the treatment of cancer. This study was performed to clarify the effects of evodiamine in mice tumor model studies.

**Methods:**

Electronic databases and search engines involved China Knowledge Resource Integrated Database (CNKI), Wanfang Database, Chinese Scientific Journal Database (CSJD-VIP), China Biomedical Literature Database (CBM), PubMed, Embase, Web of Science, and ClinicalTrials.gov databases, which were searched for literature related to the antitumor effects of evodiamine in animal tumor models (all until 1 October 2021). The evodiamine effects on the tumor volume and tumor weight were compared between the treatment and control groups using the standardized mean difference (SMD).

**Results:**

Evodiamine significantly inhibited tumor growth in mice, as was assessed with tumor volume [13 studies, n=267; 138 for EVO and 129 for control; standard mean difference (SMD)= -5.99; 95% (CI): -8.89 to -3.10; I^2^ = 97.69%, *p ≤* 0.00], tumor weight [6 studies, n=89; 49 for EVO and 40 for control; standard mean difference (SMD)= -3.51; 95% (CI): -5.13 to -3.90; I^2^ = 83.02%, *p ≤* 0.00].

**Conclusion:**

EVO significantly suppresses tumor growth in mice models, which would be beneficial for clinical transformation. However, due to the small number of studies included in this meta-analysis, the experimental design and experimental method limitations should be considered when interpreting the results. Significant clinical and animal studies are still required to evaluate whether EVO can be used in the adjuvant treatment of clinical tumor patients.

## Introduction

**Figure 1:**
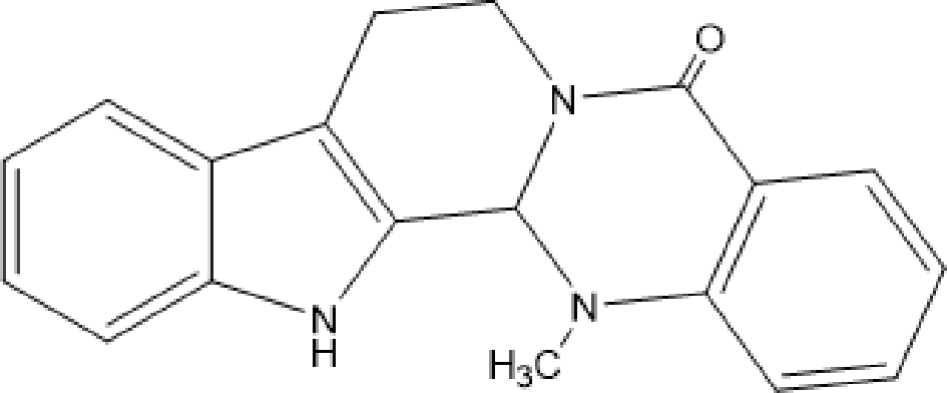
The chemical structure of evodiamine.

Evodiamine (EVO, **Figure 1**), a kind of quinazolinocarboline alkaloid, is one of the components isolated from a Chinese herbal medicine, called Wu-Chu-Yu (*Evodia rutaecarpa*) (1). It has been considered an effective Chinese medicine for the treatment of gastropathy, hypertension, and eczema (2). In recent years, the antitumor effect of EVO *in vivo* has become a focus for innovation and a hot topic in medical research. Recent work in this field suggests that the anticancer effects of EVO resist several high-risk cancers, including pancreatic cancer, lung cancer, colon cancer, lymphoma, and oral cancer (3–7). EVO, as a novel occurring indole alkaloid with attractive multitargeting antiproliferative activity, has been investigated as a leading compound that possesses multitargeting profiles (8, 9). Novel boron-containing EVO derivatives were designed, which have improved the antitumor potency of the EVO and showed a good antitumor activity *in vitro* and *in vivo* by reactive oxygen species (ROS) (8).

Although many preclinical experiments have undoubtedly shown that EVO exerts an antitumor effect, less evidence, however, has established clinical associations to guide healthcare decisions. Moreover, quality assessment of these animal studies reporting the antitumor effect of EVO is lacking. A systematic review of preclinical studies (especially animal experiments) can evaluate the efficacy and mechanism of treatment, provide reliable information for drug research, and lay the foundation for future clinical research (10). To accelerate the transformation of preclinical experiments to clinical studies, a systematic review and meta-analysis would be performed. The aim of this study is to investigate the impact of EVO on a mice tumor model.

## Materials and Methods

This study was performed according to the systematic reviews of animal experiments (11) (**Supplementary Table S1**) and preferred reporting items for systematic reviews and meta-analyses (PRISMA) (12) guidelines (**Supplementary Table S2**).

### Search Strategy

We searched China Knowledge Resource Integrated Database (CNKI), Wanfang Database, Chinese Scientific Journal Database (CSJD-VIP), China Biomedical Literature Database (CBM), PubMed, Embase, Web of Science, and ClinicalTrials.gov databases to find relevant articles up to 1 October 2021 in all languages using a combination of the main search terms “evodiamine” and “Neoplasms.” The search strategy was designed to be as broad as possible. Then, the reference lists of the relevant articles were checked for additional articles. The detailed search strategy is presented in **Supplementary Table S3**.

Two authors (YC and PHB) independently reviewed the titles and abstracts. Discrepancies were resolved through discussions between the two authors. The two authors then independently analyzed the full text of the remaining articles to determine the final inclusion.

### Selection Criteria

The inclusion criteria were as follows—(1) types of animals: mice; (2) types of intervention: EVO only; (3) type of outcome measure: the effects of EVO in mice cancer models, including tumor volume and tumor weight; and (4) study design: experiments should be prospectively controlled. The exclusion criteria were as follows: (1) review, conference reports, or editorial articles; (2) repeatedly published studies; (3) articles with incomplete data; and (4) articles with no use of experimental animals.

### Data Extraction

Two authors (YC and PHB) extracted data from the selected trials. This process was verified by another investigator (LJ). An Excel database was used to extract the following information from the included studies: (1) author name, year of publication, and country; (2) animal species, sex, and sample size; (3) methods of inducing tumor; and (4) EVO treatment conditions including administration route, dosage, frequency, duration, and types of tumor. To obtain the data not presented in the original articles, we sent two e-mails 1 week apart to the corresponding authors.

Data of all outcomes including sample size of animals (N), means, standard deviation (SD), or standard error of the mean (SEM) were extracted. If the data were presented in the form of graph, data extraction would be performed using digital image analysis software (Getdata.Ink). SEM was converted to SD by a mathematical formula: SD=SEM×√N.

### Assessment of the Methodological Quality

Each study was evaluated according to the Systematic Review Centre for Laboratory Animal Experimentation (SYRCLE) for animal model studies (13). The tool, which aims to assess methodological quality, was adapted to appraise bias in animal studies, including random sequence generation (selection bias), baseline characteristics (selection bias), allocation concealment (selection bias), random housing (performance bias), blinding of participants and personnel (performance bias), random selection of animals in the evaluation results (detection bias), blinding of outcome assessment (detection bias), incomplete outcome data (attrition bias), selective reporting (reporting bias), and other biases. Two trained researchers (YC and PHB) independently evaluated and cross-checked the inherent risk of bias in the included studies. Differences in opinion were negotiated or settled by a third party (LJ). Answers to the assessment questions (tools) were either “yes” to indicate a low risk of bias or “no” to indicate a high risk of bias. An answer of “unclear” was assigned to items for which a “yes” or “no” answer was not clear.

### Statistical Analysis

We performed the analysis with use of STATAMP, version 16. For the outcome measures of tumor volume and tumor weight, we will use standard mean difference (SMD). Considering the variation between studies, the random-effects model was used at a 95% confidence level. In addition, the heterogeneity of the studies was assessed by forest plots using I^2^ statistics. Then, a subgroup analysis was performed based on gender, mode of administration, the types of tumor, and animal species. A p-value of less than 0.05 was considered significant. Funnel plots were performed to analyze publication bias. A sensitivity analysis was conducted only when three or more studies were included in the comparison. Sensitivity analysis was used to assess the robustness of meta-analysis results by sequentially removing individual included studies. The presence of the publication bias was checked for each outcome through funnel plots. Publication bias was assessed using the Egger regression asymmetry test when the comparison contained at least 10 studies (14).

## Results

### Selection of Studies

**Figure 2:**
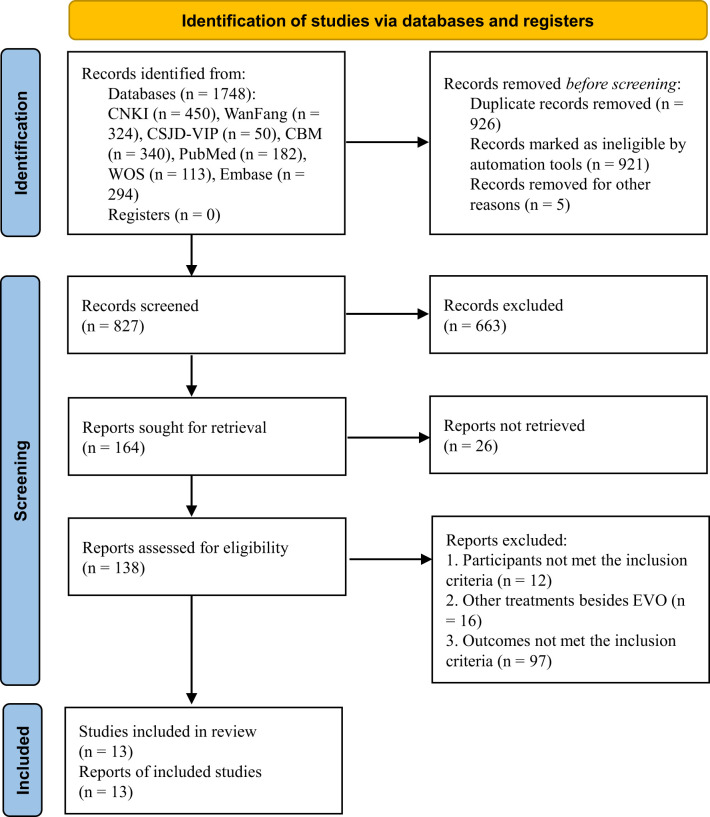
Flow of the selection of studies.

A total of 1,748 studies were identified in the electronic database search. After selection, 13 studies (1, 3, 4, 6, 15–23) met the inclusion criteria and were included in our meta-analysis. The flow of literature screening is shown in **Figure 2**.

### Study Characteristics

Study characteristics are presented in **Table 1**. A total of 13 studies from 2012 to 2021 met the inclusion criteria, containing various cancer types, including lymphoma, oral cancer, colon cancer, hepatocellular cancer, ovarian cancer, lung cancer, and pancreatic cancer. The studies all used mice, and the mice were modeled *via* subcutaneous tumor implantation or induced tumor formation. EVO was administered in doses ranging between 3 and 100 mg/kg body weight through subcutaneous injection, intraperitoneal injection, and gavage. The size of the study sample ranged from 4 to 18, while the course of treatment ranged from 14 to 70 days.

**Table 1:** Characteristics of included studies.

Author, year	Sample	Types of tumor	Tumor Model	Mode of administration	Dose	Duration	N (M/E)	Outcomes
Wei et al., 2012 (20)	Nude Balb/c Mice, F	Pancreatic cancer	Xenograft model	Intraperitoneal injection	10 mg/kg/d	37 d	24 (12/12)	TW; TV
Li et al., 2014 (18)	Nude Balb/c Mice, F	Oral squamous cell carcinoma	Xenograft model	Gavage	10 mg/kg/d	28 d	8 (4/4)	TW; TV
Lee et al., 2015 (17)	SCID Mice, NA	Ovarian cancer	Xenograft model	Intraperitoneal injection	100 mg/kg	NA	10 (5/5)	TV
Shi et al., 2017 (19)	Nude Balb/c Mice, F	Colorectal carcinoma	Xenograft model	Gavage	3 mg/kg/d	22 d	30 (15/15)	TV
Yang et al., 2017 (21)	Balb/c Mice, M	Lymphoma	Xenograft model	Gavage	10 mg/kg/d	24 d	32 (16/16)	TV
Hu et al., 2017 (1)	C57BL/6 Mice, M	Liver cancer	Xenograft model	Fed	20 mg/kg, 3d	30 d	40 (20/20)	TV
Guo et al., 2018 (15)	Nude Balb/c Mice, NA	Liver cancer	Xenograft model	NA	10 mg/kg/d	21 d	12 (6/6)	TW; TV
Guo et al., 2019 (4)	Nude Balb/c Mice, M	Oral squamous cell carcinoma	Xenograft model	Intraperitoneal injection	10 mg/kg/d	35 d	12 (6/6)	TW; TV
Jiang et al., 2020 (6)	Balb/c Mice, F	Lung cancer	Xenograft model	Gavage	20 mg/kg/d	22 d	6 (3/3)	TW; TV
Deng et al., 2020 (3)	KM Mice, M	Lymphoma	Xenograft model	Gavage	20 mg/kg, 3d	21 d	10 (5/5)	TV
Zeng et al., 2021 (23)	Nude Balb/c Mice, F	Colorectal carcinoma	Xenograft model	Intraperitoneal injection	10 mg/kg/d	22 d	20 (10/10)	TV
Hyun et al., 2021 (16)	NOD/SCID Mice, NA	Lung cancer	Xenograft model	Gavage	20 mg/kg/d	14 d	27 (18/9)	TW; TV
Zhu et al., 2021 (22)	C57 Mice, NA	Colorectal carcinoma	Drug-induced model	NA	10 mg/kg/d	70 d	16 (8/8)	TV

F, female; M, male; NA, not applicable; d, day; N(M/E), number (model/evodiamine group); TW, tumor weight; TV, tumor volume.

### Quality Assessment

**Figure 3:**
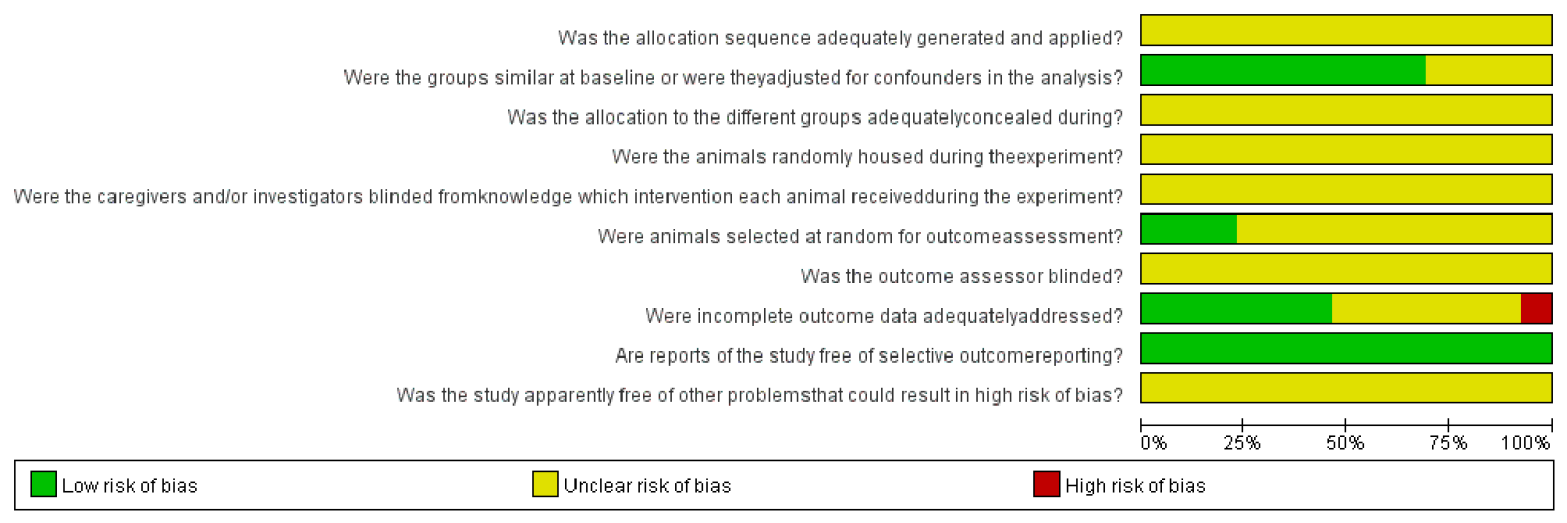
The risk of bias graph of included studies.

The quality assessment results of the 13 included studies are shown in **Figure 3** and **Supplementary Figure S1**. All of the studies did not report the following aspects: random sequence generation, allocation concealment, random housing, blinding of participants and personnel, random selection of animals in the evaluation results, and blinding of outcome assessment. Baseline characteristics were not fully reported in most studies. One study showed high risk of bias on the attrition bias item of incomplete outcome data with the main problem of an unclear number of animals. Although the overall quality of the studies was poor, none of the papers were excluded because of their quality or risk of bias assessment.

### Tumor Volume

**Figure 4:**
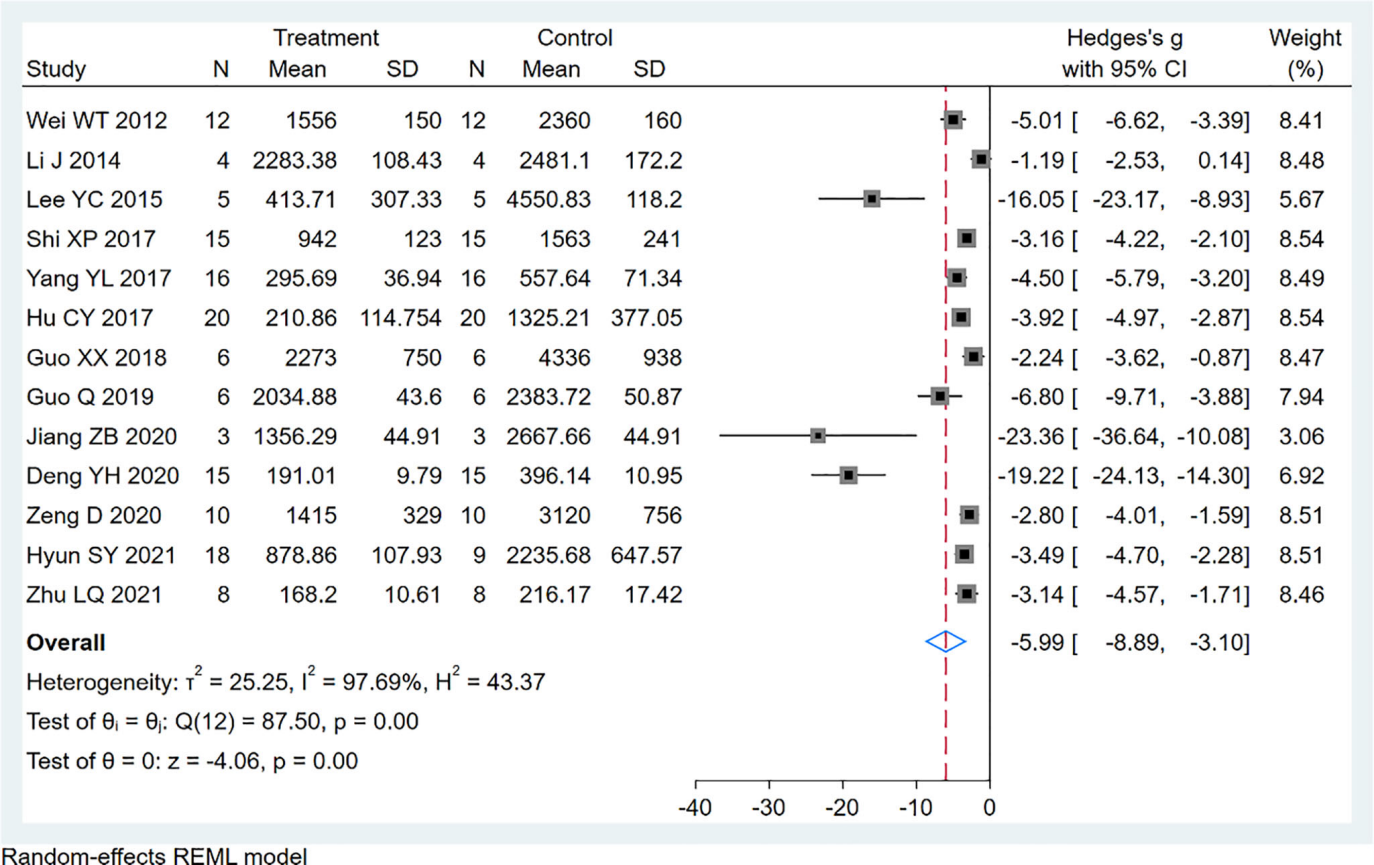
Forest plot of tumor volume.

Thirteen studies using xenograft models or an induced tumor model were included to investigate the effect of EVO treatment on tumor volume (**Figure 4**). A total of 138 mice were in the intervention group, while 129 mice were in the control group. The pooled estimate suggested a significant inhibition of tumor volume [SMD -5.99; 95% CI -8.89, -3.10; P ≤ 0.00]. The heterogeneity among studies was relatively high (I^2^ = 97.69%, P ≤ 0.00). Subgroup analysis had not found the sources of the significant heterogeneity (**Table 2**).

**Table 2:** Sub-analysis of the effects of EVO on tumor volume.

Variable	Tumor volume			
	Number of studies	95% CI	*P*-value	I^2^ (%)
Overall	13	-5.99 (-8.89,-3.10)	≤0.00	97.69
Types of tumor				
Pancreatic cancer	1	-5.01 (-6.62,-3.39)	─	─
Oral squamous cell carcinoma	2	-3.84 (-9.32,1.64)	≤0.00	91.47
Ovarian cancer	1	-16.05 (-23.17,-8.93)	─	─
Colorectal carcinoma	3	-3.04 (-3.73,-2.34)	0.90	0
Lymphoma	2	-11.66 (-26.08,2.76)	≤0.00	96.90
Liver cancer	2	-3.14 (-4.78,-1.50)	0.06	72.2
Lung cancer	2	-11.66 (-26.08,2.76)	≤0.00	88.28
Species				
Mice	7	-9.29 (-15.10,-3.49)	≤0.00	98.62
Nude mice	6	-3.28 (-4.66,-1.91)	≤0.00	81.66
Gender				
Female	5	-3.29 (-4.90,-1.68)	≤0.00	80.21
Male	4	-8.30 (-14.99,-1.62)	≤0.00	98.17
NA	4	-5.33 (-10.45,-0.21)	≤0.00	97.30
Mode of administration				
Intraperitoneal injection	4	-6.73 (-11.31,-2.16)	≤0.00	94.24
Gavage	6	-7.91 (-14.54,-1.28)	≤0.00	99.01
Fed	1	-3.92 (-4.97,-2.87)	─	─
NA	2	-2.68 (-3.67,-1.68)	0.37	0

NA, not applicable.

### Tumor Weight

**Figure 5:**
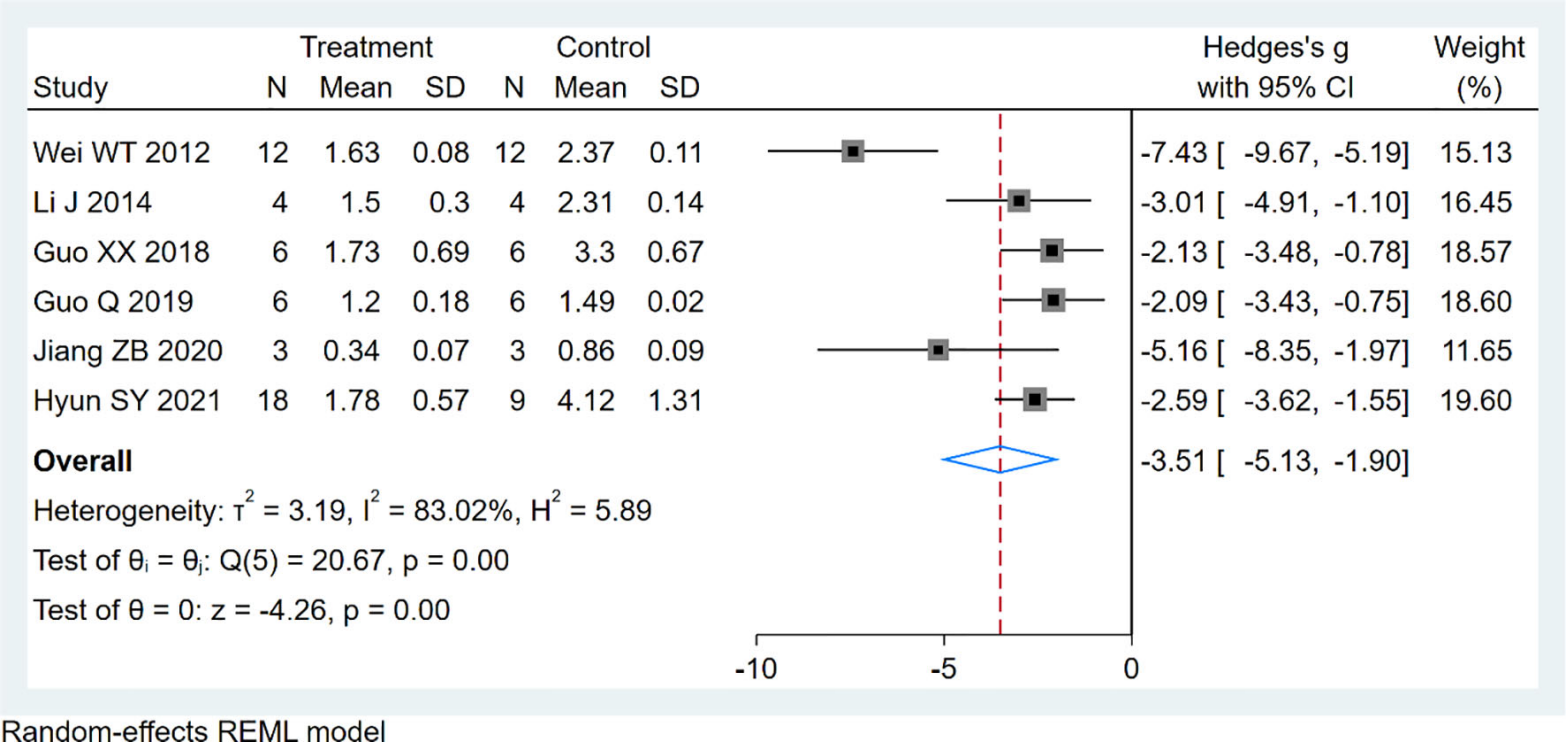
Forest plot of tumor weight.

Six studies using xenograft models were included to investigate the effect of EVO treatment on tumor weight (**Figure 5**). A total of 95 mice were in the intervention group, while 95 mice were in the control group. The pooled estimate suggested a significant inhibition of tumor weight [SMD -3.51; 95% CI -5.13, -1.90; P ≤ 0.00]. The heterogeneity among studies was relatively high (I^2^ = 83.02%, P ≤ 0.00). Subgroup analysis had not found the sources of the significant heterogeneity (**Table 3**).

**Table 3:** Sub-analysis of the effects of EVO on tumor weight.

Variable	Tumor weight			
	Number of studies	95% CI	*P*-value	I^2^ (%)
Overall	6	-3.51 (-5.13,-1.90)	≤0.00	83.02
Types of tumor				
Pancreatic cancer	1	-7.43 (-9.67,-5.19)	─	─
Oral squamous cell carcinoma	2	-2.39 (-3.49,-1.30)	0.44	0
Liver cancer	1	-2.13 (-3.48,-0.78)	─	─
Lung cancer	2	-3.41 (-5.77,-1.06)	0.13	55.84
Species				
Mice	2	-3.41 (-5.77,-1.06)	0.13	55.84
Nude mice	4	-3.55 (-5.92,-1.18)	≤0.00	87.96
Gender				
Female	3	-5.15 (-7.86,-2.44)	0.01	74.04
Male	1	-2.09 (-3.43,-0.75)	─	─
NA	2	-2.42 (-3.24,-1.59)	0.6	0
Mode of administration				
Intraperitoneal injection	2	-4.68 (-9.91,0.55)	≤0.00	93.79
Gavage	3	-2.87 (-3.75,-1.99)	0.32	0
NA	1	-2.13 (-3.48,-0.78)	─	─

NA, not applicable.

### Sensitivity Analysis

**Supplementary Figures S2, S3** show that the results of tumor volume and tumor weight were robust.

### Publication Bias

**Figure 6:**
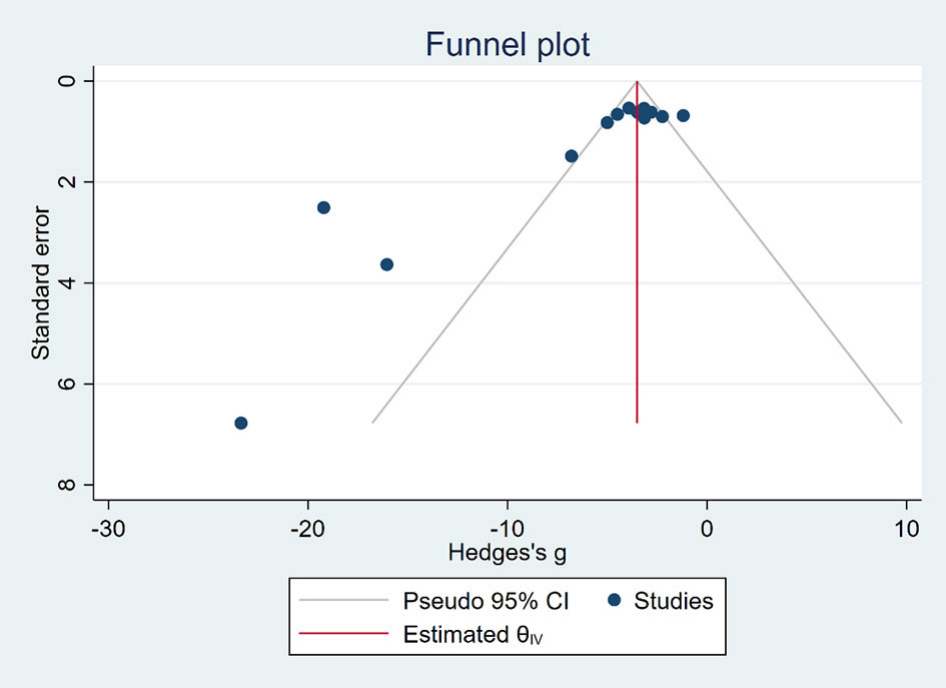
Funnel plot of tumor volume.

**Figure 7:**
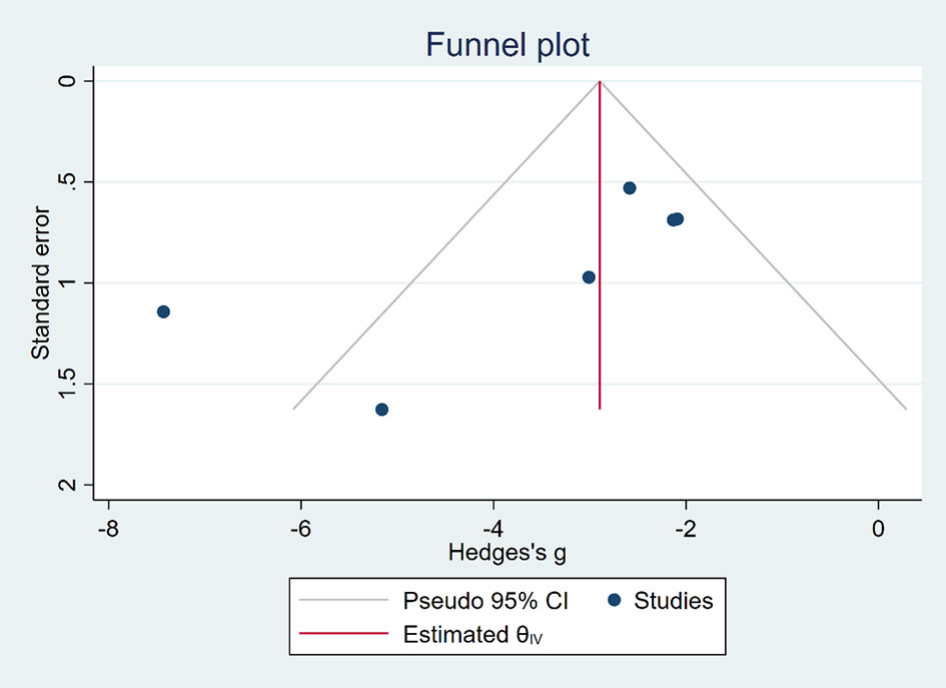
Funnel plot of tumor weight.

Funnel plots (**Figures 6**, **7**) were used to express publication bias. The standard error was plotted against the tumor volume and tumor weight (13 publications evaluating the inhibitory effects of EVO on tumor volume and 6 on tumor weight). Visual inspection of the funnel plot test suggested that publication bias was possible. In this research, we were unable to evaluate publication bias because some positive results may be much easier to be published.

## Discussion

This meta-analysis was conducted to evaluate the effects of EVO on animal tumor models. It suggested that EVO generates a significant antitumor activity in animal models of tumor. It has been shown that EVO mediates tumor apoptosis mainly through a mitochondrial pathway (24, 25). EVO could increase the ratio of BAX/BCL to sensitize cells to death signals (15, 20, 26). Then, it would activate apoptotic initiation protease caspase-9 and its downstream apoptotic execution protease caspase-3 (27–30). Also, the key rate-limiting step of apoptosis release of cyto-C was observed (28). Moreover, EVO suppresses cancer growing by eliminating stemness and inhibiting invasion and migration (7, 31). This is mainly due to the downregulation of epithelial-mesenchymal transition (EMT) (32–34). For instance, EVO elevated epithelial marker E-cadherin and reduced the expression of mesenchymal markers N-cadherin and vimentin (33), as well as matrix metalloproteinase-2 (MMP-2) and matrix metalloproteinase-9 (MMP-9) protein levels (32).

Many *in vitro* and *in vivo* studies had reported beneficial effects of EVO against tumor, and as far as we know, this is the first meta-analysis that assessed the anticancer effect of EVO in preclinical experiments. Our results showed that both the tumor volume and tumor weight results of mice animal models treated with EVO showed significant improvement, indicating that EVO can probably be used as a new treatment strategy in the treatment of clinical tumor patients.

Although this study achieved positive results, the possible shortcomings should not be ignored. First, our research only includes the available data; some negative results are less likely to have been published. Therefore, this meta-analysis may have exaggerated the effect size. Second, the heterogeneity among the various animal studies was quite considerable, as animal studies are often explorative and heterogeneous compared with clinical trials. As a consequence, a subgroup analysis involving cancer types, species, mode of administration, and gender was conducted; however, due to the limited sample size of each subgroup and insufficient statistical power, it did not find the source of significant heterogeneity. Heterogeneity might have originated from different animal ages, varied administration routes, and different initiation times for treatment. Third, our study still lacks the findings of the effect of EVO in tumor mice models with specific types of diseases. Clinical patients are more likely to present with different types of underlying diseases, so there is still a lot of work to be done in clinical translation. In addition, we could not get exact conclusion in this condition: the treatment methods in control arms did not unify to an extent; the numbers of animals varied from group to group.

## Conclusions

EVO appears to have the highest efficacy in reducing tumor volume and weight, which increases our confidence in the results and their transformation to the clinical situation. This is a result of antiproliferative, anti-invasive, and apoptosis-inducing functions of EVO in animal experiments. However, due to the small number of studies included in this meta-analysis, the experimental design and experimental method limitations should be considered when interpreting the results. Significant clinical and animal studies are still required to evaluate whether EVO can be used in the adjuvant treatment of clinical tumor patients.

## Data Availability Statement

The original contributions presented in the study are included in the article/**Supplementary Material**. Further inquiries can be directed to the corresponding authors.

## Author Contributions

YC, LJ, and CKL contributed to conception and design of the study. YC and CJ organized the database. YC, CJ, PHB, and YSJ performed the statistical analysis. YC, CJ, and LJ wrote the first draft of the manuscript. All authors contributed to manuscript revision, read, and approved the submitted version.

## Funding

This study was supported by Key project at central government level: The ability establishment of sustainable use for valuable Chinese medicine resources (2060302); The Opening Project of Hubei Provincial Key Laboratory of Occurrence and Intervention of Rheumatic Diseases (Hubei Minzu University)(PT022002); The Opening Project of Zhejiang Provincial Preponderant and Characteristic Subject of Key University (Traditional Chinese Pharmacology), Zhejiang Chinese Medical University (No. ZYAOXYB2019003).

## Conflict of Interest

The authors declare that the research was conducted in the absence of any commercial or financial relationships that could be construed as a potential conflict of interest.

## Publisher’s Note

All claims expressed in this article are solely those of the authors and do not necessarily represent those of their affiliated organizations, or those of the publisher, the editors and the reviewers. Any product that may be evaluated in this article, or claim that may be made by its manufacturer, is not guaranteed or endorsed by the publisher.
