# Supplementary material for: Antitumor Effects of Evodiamine in Mice Model Experiments: A Systematic Review and Meta-Analysis
Source: Front Oncol. 2021 Nov 9;11:774201. doi: 10.3389/fonc.2021.774201 (PMC8660089; doi:10.3389/fonc.2021.774201)
Supplement: Supplementary file 1 [file DataSheet_1.pdf]

Anti-tumor effects of evodiamine in mice model experiments: a systematic review and meta-analysis

Cong Yin[§], Jing Cheng[§], Hongbing Peng, Shijun Yuan, Keli Chen*, Li Juan*

Hubei Province Key Laboratory of Traditional Chinese Medicine Resource and Chemistry; Hubei University of Chinese Medicine, Wuhan, Hubei, China.

Supplementary materials

Table S1. the reporting checklist of systematic reviews of animal experiments.

Heading	Subheading	Descriptor	Pages
**Title**		Identify the report as a meta-analysis [or systematic review] of animal toxicology experiments	1
**Abstract**	Objectives	Use a structured format<br>Describe explicitly the scientific question/ hypothesis	1
	Data sources	Describe the databases and other important information sources used	1
	Review methods	Describe the selection criteria (e.g. species, strain, intervention/exposure, outcome and study design): methods for validity assessment and data abstraction, the experiment characteristics, and quantitative data synthesis methods	1
	Results	Describe characteristics of the experiments included and excluded; qualitative and quantitative findings (e.g. point estimates and confidence intervals/standard errors), stating clearly what is estimated: dose-response curves, LD50 etc; and subgroup analyses	1
	Conclusion	State the main results and their implications	1
**Introduction**		Describe the scientific problem explicitly, biological rationale for the intervention/exposure, and rationale for the review	2
**Methods**	Searching	Describe the information sources in detail (e.g. databases, registers, personal files, expert informants, agencies, hand-searching), including keywords, search strategy and any restrictions (years considered, publication status, language of publication)<br>Describe special efforts to include all available data (e.g. contact with authors, searching the grey literature)	2-3
	Selection	Describe the inclusion and exclusion criteria (defining intervention/exposure, principal outcomes, and experimental design)<br>List excluded experiments and reasons for exclusion	3
	Validity and quality assessment	Describe the criteria and process used (e.g. blind assessments, quality assessment, and their findings)	3-4
	Data abstraction	Describe the process or processes used (e.g. completed independently, in duplicate), including details on reproducibility,	3-4

		inter-rate agreement.<br>Whether aggregate data or individual animal data are abstracted	
	Study characteristics	Describe the type of study designs, animals' characteristics (e.g. species, strain, age, sex), details of intervention/exposure (including route of administration, dose and duration), outcome definitions	3
	Quantitative data synthesis	Describe the principal measures of effect, method of combining results (e.g. fixed- and random-effects; meta-regression), handling of missing data; how statistical heterogeneity was assessed; how data from different species and strains were dealt with; adjustment for possible confounding variables; rationale for any a-priori sensitivity and subgroup analyses; and any assessment of publication bias—all in enough detail to allow replication	3
**Results**	Flow chart	Provide a meta-analysis profile summarizing experiment flow giving total number of experiments in the meta-analysis	8
	Study characteristics	Present descriptive data for each experiment (e.g. species, strain, age, sex, sample size, intervention/exposure, dose, duration)	9
	Quantitative data synthesis	Report agreement on the selection and validity of assessment and relevance to the scientific question/hypothesis; present simple summary results (e.g. forest plot); present data needed to calculate effect sizes and confidence intervals; identify sources of heterogeneity, impact of study quality and publication bias	5-6
**Discussion**		Summarize key findings; discuss scientific/clinical inferences and generalizability based on internal and external validity; interpret the results in light of the totality of available evidence, including data from human studies; discuss rationale for use of animal data to help inform human health outcomes; critically appraise potential biases in the review process (e.g. publication bias); suggest a future research agenda	6-7

Table S2. the reporting checklist of systematic reviews and meta-analyses (PRISMA).

Section and Topic	Item #	Checklist item	Location where item is reported
**TITLE**			
Title	1	Identify the report as a systematic review.	1
**ABSTRACT**			
Abstract	2	See the PRISMA 2020 for Abstracts checklist.	1
**INTRODUCTION**			
Rationale	3	Describe the rationale for the review in the context of existing knowledge.	2
Objectives	4	Provide an explicit statement of the objective(s) or question(s) the review addresses.	2
**METHODS**			
Eligibility criteria	5	Specify the inclusion and exclusion criteria for the review and how studies were grouped for the syntheses.	2-3
Information sources	6	Specify all databases, registers, websites, organisations, reference lists and other sources searched or consulted to identify studies. Specify the date when each source was last searched or consulted.	2-3
Search strategy	7	Present the full search strategies for all databases, registers and websites, including any filters and limits used.	Supplementary materials 8-9
Selection process	8	Specify the methods used to decide whether a study met the inclusion criteria of the review, including how many reviewers screened each record and each report retrieved, whether they worked independently, and if applicable, details of automation tools used in the process.	3
Data collection process	9	Specify the methods used to collect data from reports, including how many reviewers collected data from each report, whether they worked independently, any processes for obtaining or confirming data from study investigators, and if applicable, details of automation tools used in the process.	3
Data items	10a	List and define all outcomes for which data were sought. Specify whether all results that were compatible with each outcome domain in each study were sought (e.g. for all measures, time points, analyses), and if not, the methods used to decide which results to collect.	3
	10b	List and define all other variables for which data were sought (e.g. participant and intervention characteristics, funding sources). Describe any assumptions made about any missing or unclear information.	3
Study risk of bias	11	Specify the methods used to assess risk of bias in the included studies, including details of the tool(s) used,	3-4

Section and Topic	Item #	Checklist item	Location where item is reported
assessment		how many reviewers assessed each study and whether they worked independently, and if applicable, details of automation tools used in the process.	
Effect measures	12	Specify for each outcome the effect measure(s) (e.g. risk ratio, mean difference) used in the synthesis or presentation of results.	3-4
Synthesis methods	13a	Describe the processes used to decide which studies were eligible for each synthesis (e.g. tabulating the study intervention characteristics and comparing against the planned groups for each synthesis (item #5)).	3-4
	13b	Describe any methods required to prepare the data for presentation or synthesis, such as handling of missing summary statistics, or data conversions.	3-4
	13c	Describe any methods used to tabulate or visually display results of individual studies and syntheses.	3-4
	13d	Describe any methods used to synthesize results and provide a rationale for the choice(s). If meta-analysis was performed, describe the model(s), method(s) to identify the presence and extent of statistical heterogeneity, and software package(s) used.	3-4
	13e	Describe any methods used to explore possible causes of heterogeneity among study results (e.g. subgroup analysis, meta-regression).	3-4
	13f	Describe any sensitivity analyses conducted to assess robustness of the synthesized results.	3-4
Reporting bias assessment	14	Describe any methods used to assess risk of bias due to missing results in a synthesis (arising from reporting biases).	3-4
Certainty assessment	15	Describe any methods used to assess certainty (or confidence) in the body of evidence for an outcome.	3-4
**RESULTS**			
Study selection	16a	Describe the results of the search and selection process, from the number of records identified in the search to the number of studies included in the review, ideally using a flow diagram.	4-5
	16b	Cite studies that might appear to meet the inclusion criteria, but which were excluded, and explain why they were excluded.	4-5
Study characteristics	17	Cite each included study and present its characteristics.	9
Risk of bias in studies	18	Present assessments of risk of bias for each included study.	10

Section and Topic	Item #	Checklist item	Location where item is reported
Results of individual studies	19	For all outcomes, present, for each study: (a) summary statistics for each group (where appropriate) and (b) an effect estimate and its precision (e.g. confidence/credible interval), ideally using structured tables or plots.	Supplementary materials 10
Results of syntheses	20a	For each synthesis, briefly summarise the characteristics and risk of bias among contributing studies.	5-6
	20b	Present results of all statistical syntheses conducted. If meta-analysis was done, present for each the summary estimate and its precision (e.g. confidence/credible interval) and measures of statistical heterogeneity. If comparing groups, describe the direction of the effect.	5-6
	20c	Present results of all investigations of possible causes of heterogeneity among study results.	5-6
	20d	Present results of all sensitivity analyses conducted to assess the robustness of the synthesized results.	5-6
Reporting biases	21	Present assessments of risk of bias due to missing results (arising from reporting biases) for each synthesis assessed.	5-6
Certainty of evidence	22	Present assessments of certainty (or confidence) in the body of evidence for each outcome assessed.	5-6
**DISCUSSION**			
Discussion	23a	Provide a general interpretation of the results in the context of other evidence.	6-7
	23b	Discuss any limitations of the evidence included in the review.	6-7
	23c	Discuss any limitations of the review processes used.	6-7
	23d	Discuss implications of the results for practice, policy, and future research.	6-7
**OTHER INFORMATION**			
Registration and protocol	24a	Provide registration information for the review, including register name and registration number, or state that the review was not registered.	NA
	24b	Indicate where the review protocol can be accessed, or state that a protocol was not prepared.	NA
	24c	Describe and explain any amendments to information provided at registration or in the protocol.	NA
Support	25	Describe sources of financial or non-financial support for the review, and the role of the funders or sponsors in the review.	7
Competing interests	26	Declare any competing interests of review authors.	7

Section and Topic	Item #	Checklist item	Location where item is reported
Availability of data, code and other materials	27	Report which of the following are publicly available and where they can be found: template data collection forms; data extracted from included studies; data used for all analyses; analytic code; any other materials used in the review.	7

Table S3. Chinese and English search strategies

Comments:

In order for non-Chinese readers to understand the Chinese search strategy of this article, we translated the Chinese search terms in the search formula.

CNKI	(SU=吴茱萸碱) AND (SU=癌 OR SU=肿瘤)<br>(SU=Evodiamine) AND (SU=Cancer OR SU=Tumor)
WanFang Database	主题:(吴茱萸碱) AND 主题:(癌 OR 肿瘤)<br>Subject:(Evodiamine) AND Subject:(Cancer OR Tumor)
CSJD-VIP	M=(吴茱萸碱) AND M=(癌 OR 肿瘤)<br>M=(Evodiamine) AND M=(Cancer OR Tumor)
CBM	("吴茱萸碱"[常用字段:智能]) AND ("癌"[常用字段:智能] OR "肿瘤"[常用字段:智能])<br>("Evodiamine"[common field: smart]) AND ("Cancer"[common field: smart] OR " Tumor"[common field: smart])
PubMed	(((Evodiamine[Supplementary Concept]) OR (Evodiamine[Title/Abstract])) OR (Isoevodiamine[Title/Abstract])) AND ((((((((((((((Neoplasms[MeSH Terms]) OR (Neoplasms[Title/Abstract])) OR (Neoplasia[Title/Abstract])) OR (Neoplasias[Title/Abstract])) OR (Neoplasm[Title/Abstract])) OR (Tumors[Title/Abstract])) OR (Tumor[Title/Abstract])) OR (Cancer[Title/Abstract])) OR (Cancers[Title/Abstract])) OR (Malignancy[Title/Abstract])) OR (Malignancies[Title/Abstract])) OR (Malignant Neoplasms[Title/Abstract])) OR (Malignant Neoplasm[Title/Abstract])) OR (Benign Neoplasms[Title/Abstract])) OR (Benign Neoplasm[Title/Abstract]))
Web of Science	#3 #1 AND #2<br>#2 TS=(Neoplasms OR Neoplasia OR Neoplasias OR Neoplasm OR Tumors OR Tumor OR Cancer OR Cancers OR Malignancy OR Malignancies OR Malignant Neoplasms OR Malignant Neoplasm OR Benign Neoplasms OR Benign Neoplasm)<br>#1 TS=(Evodiamine OR Isoevodiamine)

Embase	#7 #3 AND #6<br>#6 #4 OR #5<br>#5 Neoplasms:ab,ti,kw OR Neoplasia:ab,ti,kw OR Neoplasias:ab,ti,kw OR Neoplasm:ab,ti,kw OR Tumors:ab,ti,kw OR Tumor:ab,ti,kw OR Cancer:ab,ti,kw OR Cancers:ab,ti,kw OR Malignancy:ab,ti,kw OR Malignancies:ab,ti,kw OR 'Malignant Neoplasms':ab,ti,kw OR 'Malignant Neoplasm':ab,ti,kw OR 'Benign Neoplasms':ab,ti,kw OR 'Benign Neoplasm':ab,ti,kw<br>#4 'neoplasm'/exp<br>#3 #1 OR #2<br>#2 Evodiamine:ab,ti,kw OR Isoevodiamine:ab,ti,kw<br>#1 'Evodiamine'/exp

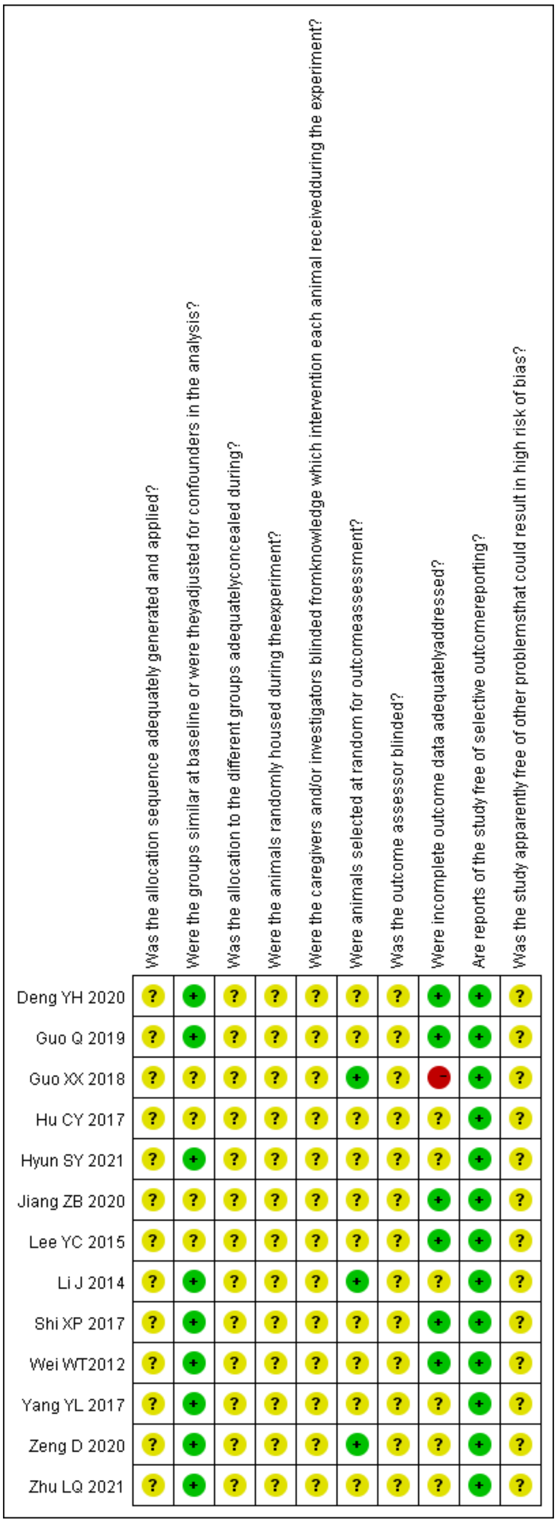

Figure S1. Risk of bias summary of included studies.

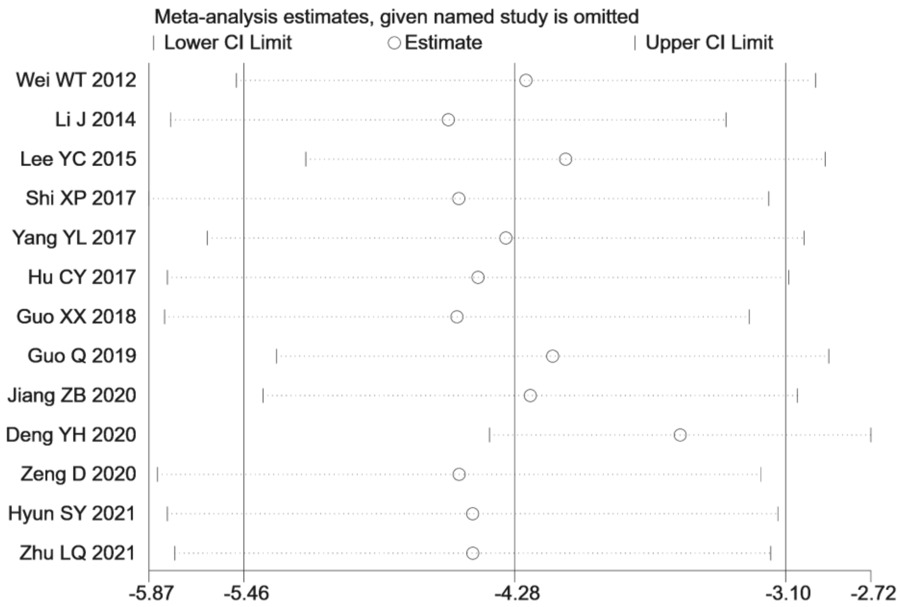

Figure S2. Sensitivity analysis of tumor volume.

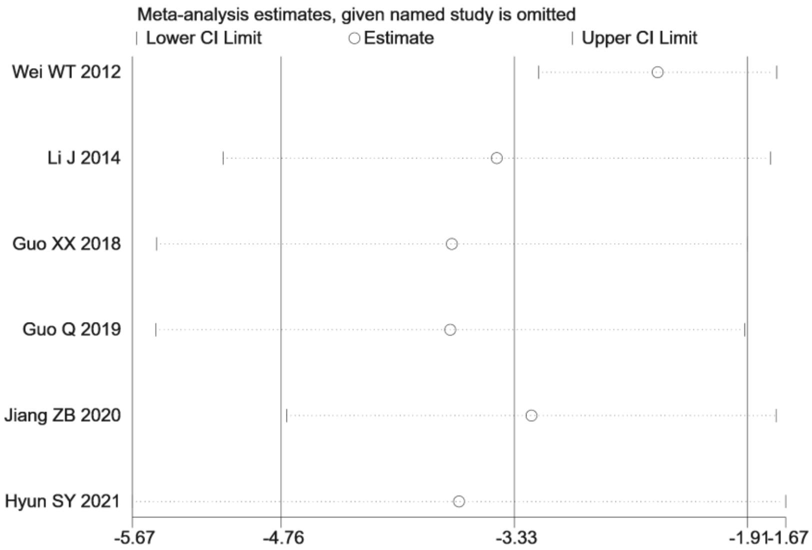

Figure S3. Sensitivity analysis of tumor weight.
